# Supplementary material for: Chromothripsis during telomere crisis is independent of NHEJ, and consistent with a replicative origin
Source: Genome Res. 2019 May;29(5):737–49. doi: 10.1101/gr.240705.118 (PMC6499312; doi:10.1101/gr.240705.118)
Supplement: Supplemental Material [file supp_gr.240705.118_Supplemental_file_1.zip › contigs/annotated_contigs/DB79/contig.2.DB79_length_663_mean_cov_14.185520362.docx]

**DB79_length_663_mean_cov_14.185520362**

```
CTTTCTTTTGTAAATTGCCCAGTTTCAGGTATGTCTTCATTAGCAGCGTGAAAATGGACTAATACATGAGGTTGCCTCTGGCATCTCCT
 >chr7:99532427-99532760 - E=1e-188
CTGTGAAGAGTCCAGCATTGACCCTGAGCACTGTCACTTCGAACACAGGAGACCAGTCCCTCTCTGCACACTCCCTCAGCCCTGTGAGT

TACTCTCCTCACTTTGCTGCTTCCCATTCTTGGCGGTATCTCTGGAGAGACTCCTGGCTCTAGCAGTGGGGCTAGGAATGGCCATGGGG

ACAGGCGTCACAGTTCTCCCTGGGCCTGACGGAGAGGGGTCTGGGTTCTCTGGATTCCTGAAGGG|A|ATATTCTGAAGCATCATTTGC
                                                                  >chr7:104474922-10447
TTATTGAAATCACCCATTTATGTCAGTGGGGTCTAGCTAAGCAGAAGAGACCAAGATGGCAAGTTCAGCTGCTGAAGTGTCGCAGTTGC
5253 + E=2e-187
CCTTGTCAAGTGGAGACAAACACATACAAAGGAGGGATGGGAGGTTGATGGAAGGAAGACATACGCCCAAAGCCAGCTCATCAACTTCC

AATTTTCTATTGACTTGTCAATTAAACACATGTGTGCACAATCCATTCAGCATGGCCCATGCAGGTCCTGCAGGCGGAGAGGGCTGCTT

CAGCTTCATGATGGTTTCGGTGGAACATGCCCAAGGGATTCC
```
